# The prevalence of herpes simplex virus type 1 and 2 infection in Iran: A meta-analysis

**Published:** 2016-10

**Authors:** Mina Malary, Ghasem Abedi, Zeinab Hamzehgardeshi, Mahdi Afshari, Mahmood Moosazadeh

**Affiliations:** 1 *Student Research Committee, School of Nursing and Midwifery, Mazandaran University of Medical Sciences, Sari, Iran.*; 2 *Department of Public Health, Health Sciences Research Center, Faculty of Health, Mazandaran University of Medical Sciences, Sari, Iran.*; 3 *Sexual and Reproductive Health Research Center, Mazandaran University of Medical Sciences, Sari, Iran.*; 4 *Department of Reproductive Health and Midwifery, Mazandaran University of Medical Sciences, Sari, Iran.*; 5 *Traditional and Complementary Medicine Research Center, Mazandaran University of Medical Sciences, Sari, Iran.*; 6 *Department of Community Medicine, Zabol University of Medical Sciences, Zabol, Iran.*; 7 *Health Sciences Research Center, Mazandaran University of Medical Sciences, Sari, Iran.*

**Keywords:** *Herpes*, *Virus*, *Meta-analysis*

## Abstract

**Background:**

Seroepidemiologic studies indicate a high prevalence of herpes simplex virus (HSV) infection. This infection leads to ophthalmic, dermatologic, oral, neurologic, vaginal and cervical problems. Different studies have been carried out to estimate the HSV seroprevalence in Iran. Combining the results of these studies would be useful for health policy-making.

**Objective:**

This study aims to estimate the pooled prevalence of HSV infection using meta-analysis.

**Materials and Methods:**

Using relevant keywords, national and international data banks were searched. Restricting the search strategy, excluding duplicates and investigating the titles and abstracts, relevant articles were identified. To increase the search sensitivity, the lists of references were investigated. To find un-published studies, specialized experts as well as research centers were interviewed. The heterogeneity between the results was assessed using Cochrane test and I-squared indicator. The pooled prevalence of HSV infection was estimated using random effects model.

**Results:**

We recruited 33 eligible papers investigated 7762 individuals. The total prevalences (95% confidence intervals) of HSV1, HSV2 and HSV infections were estimated as of 42.04% (20.9-63.1), 6.5% (4.7-8.2) and 25.7% (8.8-42.5) respectively.

**Conclusion:**

This meta-analysis showed that the HSV2 seroprevalence among Iranian people is considerably lower than HSV1 infection.

## Introduction

Herpes simplex virus (HSV) infection is increasing worldwide (1). This infection is one of the most common human pathogens with a life-time latency period within neural ganglions which can be periodically activated. Two kinds of HSV infection have been identified including HSV1 and HSV2. These kinds of infection are different regarding genetic, place of involvement, seroprevalence and re-activating rate. Both of them are spread by direct contact. HSV1 is occurred mainly during infancy, while HSV2 often involves teenagers and adults and is a sexual transmitted pathogen with painful genital lesions (2-4). In the late 1970, seroprevalence of HSV2 increased up to 30% indicating one individual out of five persons (5).

In 2012, about 417 million (11.3%) 15-49 year-old individuals were infected to HSV2 worldwide (4). Annual incidence of HSV2 infection is 23 millions. HSV1 seroprevalence has been reported more than 90% in many countries (6). During the last two decades, this infection affected from 50% to more than 85% of German, Spanish and Norwegian population (2). However, based on the National Health and Nutrition Examination Survey (NHANES), there was a reduction in the seroprevaence of HSV1 (53.9%) and HSV2 (15.7%) during 2005-2010 (7). HSV2 prevalence is associated with age, sex, number of sexual partners and socio-economic status (2). According to the HNANES reports, during 2007-2010, prevalence of HSV2 infection among men and women were 10.6% and 20.3% which was more common among non-Hispanic black women (49.9%) (8).

Oral, labial and genital infections are most common infections developed by HSV. Oral and labial infections are mainly occurred by HSV1 while, genital infections are related to both HSV1 and HSV2 (3). These infections are subclinical, therefore, most infected persons have no knowledge about their infection. These infections can rarely lead to serious complications. Both of them have perineal transmission during labor leading fatal neonatal infections. Other complications among adults are blindness, encephalitis and aseptic meningitis. HSV2 causes 2-3 folds increase in the risk of developing HIV infection (7). 

Although HSV2 is not a life-threatening infection, it may cause fulminant hepatitis among pregnant women and persistent severe infection among immunocompromised patients and even in normal immune persons (9). Age and gender are the main risk factors of HSV2 infection so that elderly and female gender increase the risk of infection. Moreover, the number of sexual partners increases the risk of developing HSV2 infection. Other prenatal risk factors of HSV2 infection are ethnicity, poverty, cocaine abuse, early sexual activity, sexual behavior and bacterial vaginosis. There is a high prevalence of genital herpes among pregnant women. HSV2 seroprevalence among pregnant Italian women has been reported from 7.6% to 8.4%. In addition, 22% of US women are infected with HSV2, 2% of which developed genital herpes during pregnancy (5). 

According to the NHANES serological data between 1988 and 2004, seroprevalences of HSV1, HSV2 and both types among pregnant women were 22%, 63% and 13% respectively (10). Genital herpes during pregnancy can lead to spontaneous abortion, intra uterus growth retardation, pre-term labor and maternal and neonatal HSV infection. Occurring HSV infection during the third trimester, increases the risk of neonatal infection from 30-50%, while corresponding figure for infections in the early pregnancy is 1%. About 85% of perinatal transmissions are occurred during intrapartum, while maternal HSV transmission during pregnancy is not common (5). 

Direct DNA virus detection can be performed using Liquid or in situ hybridization and then PCR. ELIZA can be used for serum IgG and IgM (11). Clinical management of the infection includes preventive strategies for viral transmission as well as antiviral treatment. Public education about HSV and its complications is of great importance (12). Acyclovir has been shown to be a good choice for effective treatment. Another anti-HSV2 drugs are Famcyclovir and local pencyclovir (11).

Many studies have been published regarding HSV prevalence with a great variety of the results. Combining these prevalences using meta-analysis is valuable(13, 14). This study aims to estimate the total prevalence of HSV in Iran using meta-analysis technique.

## Materials and Methods

**Search strategy**

National (SID, Iranmedex, Magiran and Irandoc) and international (PubMed, Web of Science, Google Scholar and Scopus) databanks has been searched to identify all relevant published electronic articles from each time till December 2015. The search strategy was conducted during 1-10 January 2016 using keywords such as Herpes, Cervicitis, Genital Herpes, Polymerase Chain Reaction, Herpes Simples type 1, Herpes Simplex type 2, Frequency, Prevalence, Seroepidemiological, Seroprevalence, ELIZA, PCR, HSV1, HSV2 and their Persian equivalents by two independent researchers. We also investigated all references of the articles to increase the search sensitivity. In addition, paper sources were reviewed to find non-electronic articles. Moreover, to identify grey literatures, some relevant experts and research centers were interviewed. 

**Study selection**

During a systematic and advanced search, all related papers, reports and documents were extracted. After excluding duplicates, irrelevant studies were removed investigating the titles, abstracts and full texts respectively. We also tried to explore the results of all papers in order to identify and omit any repeated findings. Study selection process was carried out by two independent researchers. In the case of any disagreement, decision making was conducted by a third researcher. 

**Quality assessment**

All selected articles were assessed using a checklist which had been applied in the previous studies (15). This checklist which was designed according to the contents of STROBE checklist included questions from the viewpoint of different methodological aspects such as sample size, type of the study, study population, sampling methods, data collection methods and tools, variables definition, statistical methodology, study objectives and illustration of the results based on the objectives (16). One score was assigned to each question and studies with at least eight scores were considered eligible for meta-analysis (15). 

**Data extraction**

All required data such as title, first author's name, type of the study, date of publication, prevalence of HSV type 1 and 2, diagnostic laboratory method, study language, sample size and sampling method were extracted from each of the eligible articles. This information were entered into the Excel spreadsheet. 

**Inclusion criteria**

All Persian and English written articles which were selected during our comprehensive search with enough quality scores reported sample size and HSV infection prevalence were included in the study.

**Exclusion criteria**

Studies did not report HSV infection prevalence and sample size, abstracts presented in congresses without full text, cases reports, cases controls and clinical trials (since cannot report a reliable estimate of prevalence) and finally, studies did not achieve the appropriate quality score were excluded from the meta analysis. 

**Statistical analysis**

Stata V.11 software was applied for statistical analysis. Standard error of the prevalence was calculated according to binomial distribution formula. The heterogeneity between the results was assessed based on Cochrane (Q) test and I square indicator. According to the degree of heterogeneity, random effect model was used to combine the point prevalences. To assess the factors influencing the heterogeneity, the effects of diagnostic method and publication date were assessed using meta-regression models. P˂0.05 was considered statistically significant. All prevalences as well as their 95% confidence intervals were illustrated by forest plots. The size of each box indicated the weight of the study and the crossed lines represented the confidence intervals.

## Results

**Figure 1:**
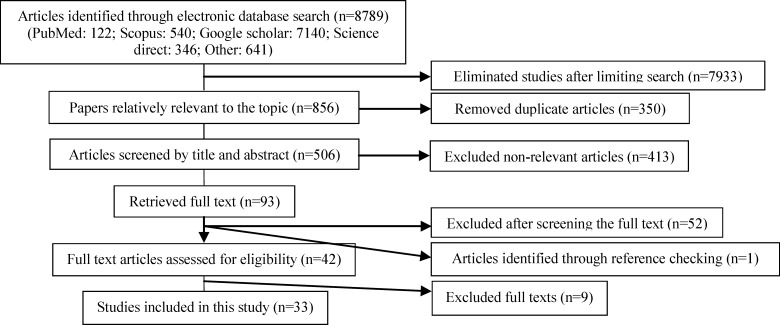
Literature search and review flowchart for selection of primary studies

During primary search, 8789 articles were identified which were limited to 850 after increased search specificity and exclusion of duplicates. Of them, 413 irrelevant papers were found after the investigation of titles and abstracts. Reviewing the full texts, 52 irrelevant articles were omitted. By checking the references, one paper was added to the list. Finally, quality assessment, excluded seven articles and 33 papers (17-49) were identified eligible for meta-analysis (Figure 1).

These studies investigated the prevalence of HSV infection among 7762 persons using ELIZA (15 studies) or PCR (16 studies). Diagnostic method did not report in two studies. Prevalence of HSV1 infection determined in 13 studies varied between 2% and 90.7% (20, 22). Prevalence of HSV2 infection reported in 18 studies from zero to 43.7% (19, 22, 45, 46). Prevalence of total HSV infection was reported in 11 studies between 3.2% and 96.1% (37, 49) (Table I).

**Figure 2:**
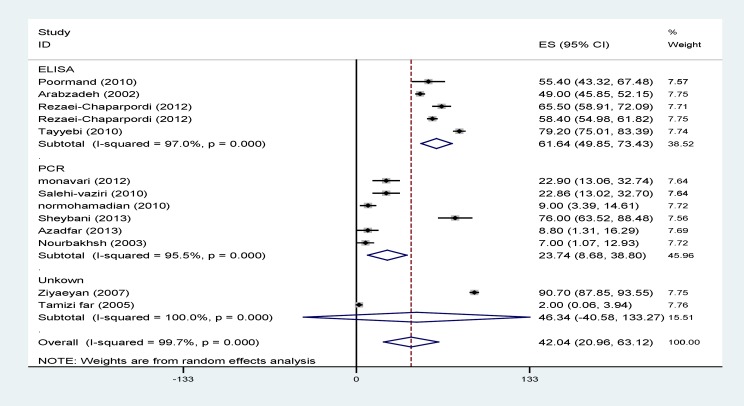
Prevalence of HSV1 according to the primary studies and diagnostic methods

**Figure 3:**
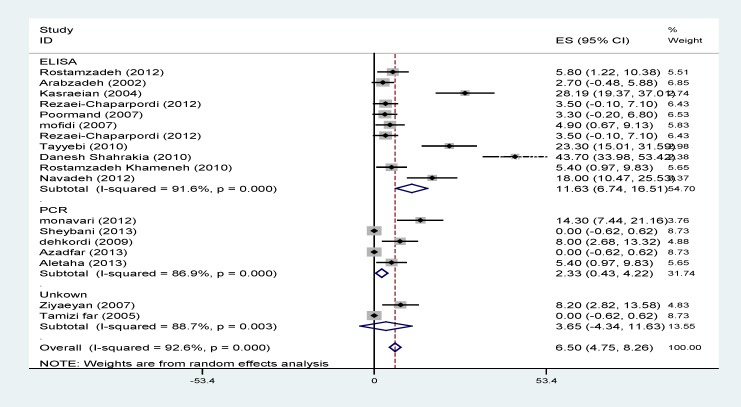
Prevalence of HSV 2 according to the primary studies and diagnostic methods

**Figure 4:**
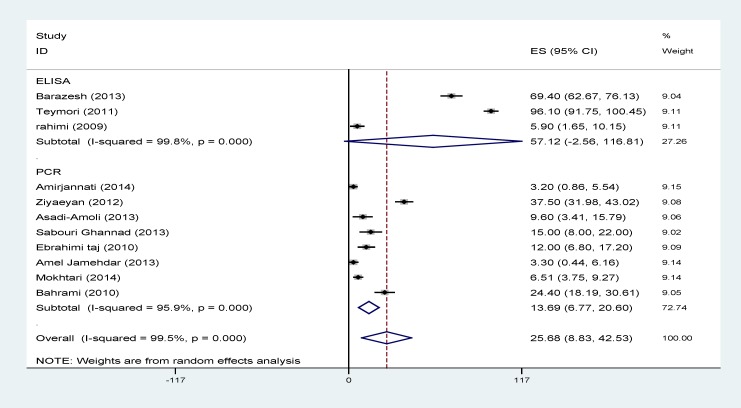
Prevalence of HSV according to the primary studies and diagnostic methods

Results of heterogeneity tests showed a great variation between the prevalence estimates of HSV. Pooled prevalences of HSV1, HSV2 and total HSV using random effect models are illustrated in table II and figures 2-4. 

The effects of publication date (β=1.7; p=0.5) and diagnostic method (β=14.01; p=0.3) on the heterogeneity were not statistically significant. The heterogeneity did not change after the subgroup analysis conducted according to the diagnostic method and publication date (Table II, Figures 2-4). 

**Table I:** Characteristics of primary studies included to the present meta-analysis

**ID**	**First author**	**Publication year**	**Measure method**	**Sample size**	**Prevalence (%)**			**Quality score**
					**HSV1**	**HSV2**	**HSV**	
1	Poormand	2010	ELISA	65	55.4	-	-	8
2	Poormand	2007	ELISA	385	-	3.3	-	10
3	Danesh Shahrakia	2010	ELISA	96	-	43.7	-	9
4	Ziyaeyan	2007	-	400	90.7	8.2	-	11
5	Rostamzadeh	2012	ELISA	86	-	5.8	-	8
6	Tamizi far	2005	-	200	2	0	-	9
7	Rostamzadeh Khameneh	2010	ELISA	91	-	5.4	-	9
8	Aletaha	2013	PCR	239	-	5.4	-	10
9	Arabzadeh	2002	ELISA	966	49	2.7	-	11
10	Bahrami	2010	PCR	184	-	-	24.4	10
11	Barazesh	2013	ELISA	180	-	-	69.4	10
12	Tayyebi	2010	ELISA	360	79.2	23.3	-	10
13	Dehkordi	2009	PCR	100	-	8	-	9
14	Ebrahimi taj	2010	PCR	150	-	-	12	10
15	Asadi-Amoli	2013	PCR	87	-	-	9.6	8
16	Kasraeian	2004	ELISA	915	-	28.19	-	11
17	Sabouri Ghannad	2013	PCR	100	-	-	15	10
18	Mofidi	2007	ELISA	406	-	4.9	-	11
19	Mokhtari	2014	PCR	307	-	-	6.51	10
20	monavari	2012	PCR	70	22.9	14.3	-	8
21	Amirjannati	2014	PCR	217	-	-	3.2	10
22	Navadeh	2012	ELISA	177	-	18	-	10
23	Nourbakhsh	2003	PCR	71	7	-	-	10
24	Rahimi	2009	ELISA	118	-	-	5.9	11
25	Rezaei-Chaparpordi	2012	ELISA	800	58.4	3.5	-	11
26	Amel Jamehdar	2013	PCR	150	-	-	3.3	10
27	Salehi-vaziri	2010	PCR	70	22.86	-	-	8
28	Rezaei-Chaparpordi	2012	ELISA	200	65.5	3.5	-	9
29	Sheybani	2013	PCR	45	76	0	-	8
30	Azadfar	2013	PCR	55	8.8	0	-	8
31	Ziyaeyan	2012	PCR	296	-	-	37.5	11
32	Normohamadian	2010	PCR	100	9	-	-	10
33	Teymori	2011	ELISA	76	-	-	96.1	9

**Table II:** The prevalence of HSV1, HSV 2 and HSV in Iran by total and subgroup analysis (The results of Meta-analysis based of random effect model

**Subgroup analysis**		**Number of study**	**Sample size**	**Prevalence (%)**	**CI**	**Heterogeneity**		
						**I-squared (%)**	**Q**	**p-value**
HSV 1								
	PCR	6	411	23.7	8.7-38.8	95.5	110.7	<0.001
	ELISA	5	2391	61.6	49.8-73.4	97	131.4	<0.001
	Total	13	3402	42.04	63.1-20.9	99.7	3587.5	<0.001
HSV 2								
	PCR	5	509	2.3	0.4-4.2	86.9	30.5	<0.001
	ELISA	11	4482	11.6	6.7-16.5	91.6	118.9	<0.001
	Total	18	5591	6.5	4.7-8.2	92.6	230.4	<0.001
HSV total								
	PCR	8	1491	13.7	6.8-20.6	95.9	170.7	<0.001
	ELISA	3	374	57.1	-2.5-116.8	99.8	870.9	<0.001
	Total	11	1865	25.7	8.8-42.5	99.5	1871.8	<0.001
HSV 1								
	<=2010	8	2232	39.4	9.6-69.2	99.8	3268.9	<0.001
	>2010	5	1170	46.2	24.1-68.3	98.1	205.2	<0.001
HSV 2								
	<=2010	10	3919	11.3	6.3-16.4	94.8	171.8	<0.001
	>2010	8	1672	3.7	1.8-5.6	87.4	55.7	<0.001
HSV total								
	<=2010	3	452	13.9	3.7-24.1	91.4	23.2	<0.001
	>2010	8	1413	30.04	7.9-52.2	99.6	1837.2	<0.001

## Discussion

Our study showed that prevalences (95% confidence intervals) of HSV1, HSV2 and total HSV among Iranian people are 25.7% (8.8-42.5), 6.5% (4.7-8.2) and 42.04% (20.9-63.1) respectively. Different studies have been carried out reporting the prevalence of infection with various types of HSV among different populations such as general population, pregnant women, blood donors, infertile patients and HIV positive persons. Similar to our results, a meta-analysis conducted in the USA reported the prevalence of HSV1 infection more than prevalence of HSV2 infection (53.9% vs. 15.7% respectively). It should be noted that in that study, HSV1 infection prevalence was decreased about 7% from 1999-2004 to 2005-2010, while, no change was occurred for HSV2 infection prevalence (7). 

In another study performed in Mexico, 3646 and 3616 adults were tested for HSV1 and HSV2 infection respectively. These prevalences were estimated as of 80.9% and 9.9% respectively increasing with age. HSV1 infection prevalence was the same between genders, while HSV2 infection was more common among women. HSV1 was more common among higher socioeconomic groups while HSV2 infection was higher among populations with low socioeconomic state (12).

According to the Lin study carried out among 2141 rural residents aged between 5-60 years in Eastern China, total prevalence of HSV1 and HSV2 infections were 92%(89.1% men and 94.2% women) and 13.2% (10.5% men and 15.3% women) respectively. Of them, 11.8% had both HSV infections. Although HSV1 and HSV2 prevalences were not differ regarding gender, coinfection of HSV1 and HSV2 was more common among women (13.6%) than in men (10%) (50). 

In a Turkish study, prevalence of HSV1 among 1072 blood samples was estimated as of 59.7% (58% for men and 61% for women with no difference) (51). In another study carried out in Pakistan among 2400 men aged 16-45 years, prevalence of HSV2 was reported as of 3.4% ranging from 1.8% in Rawalpindi to 6% in Karachi. Age more than 27 or less than 10 years, high education and more than four partners during the last 12 months were significantly associated with HSV2 (52). 

Prevalence of HSV2 infection in south of Brazil among 302 women with average age of 32.7 years using PCR was estimated as of 15.6%. The risk ratio for HIV infection using multivariate analysis was 1.9. There were no significant relationship between HSV2 infection and factors such as age of the first sexual contact, number of sexual contact, parity, gravidity, condom use and method of contraception (53). However, in a study conducted by Sierra among 869 Colombian rural women with mean age of 38±16.1 years, prevalence of HSV2 infection was reported as 19.1% strongly related to age (54). In Dubai, 6.5% of 201 women were infected with HSV infection (55).

In Burkina Faso, a study was conducted among 1674 subjects aged 15-49 years (791 men and 883 women) as well as 2018 pregnant women using ELIZA. HSV2 infection prevalence among pregnant women, men and women were 18%, 15.3% and 23.7% respectively (56). Prevalence of HSV2 infection among 487 Indian pregnant women in 2013 with mean age of 20 years in 2013 was reported as 6.7% (57). In another study conducted among 423 blood donors (54 men and 366 women) aged between 20 and 57 years in Croatia using Western Blot test, anti HSV2 antibody was positive among 3.3% of participants (2.7% men and 7% women) (58). In addition, during a different study, 39.9% of semen specimens of 69 infertile men were HSV positive (59). Comparing the seminal fluid of men with and without infertility during an analytical study, showed the frequency of HSV1 DNA virus as of 2.5% (normal semen) and 2.1% (abnormal semen) (60).

Karad in 2013 reported the prevalence of HSV2 infection among 91 Indian HIV positive patients as of 48.4%. He found that the number of sexual partners was associated with HSV2 infection. Among men, sex contact with sex workers especially before age 19 years and among women and also history of chancre were significantly correlated with positive HSV antibody. Moreover, in men, condom use was a protective factor against HSV2 infection (1). In 2012, co-infection of HSV2 and HIV infections was observed among 2.8% of Nigerian adults. In this population, prevalence of HSV2 IgG was reported as of 24.4% which was significantly higher among women compared to men (61). In Sudan, patients received kidney transplant, had higher rates of HSV antibodies in compare with control group (62). Comparing the results reported in different countries with those estimated in the current meta-analysis, indicated that the prevalence of HSV in Iran is lower than that in the other regions. 

One of the limitations in the current study is the high variation between study populations. Because of low available information about these populations, subgroup met-analysis was not conducted. The main strength of this meta-analysis was the reliable estimate of the HSV infection prevalence due to the increased power of the study which can be effectively used for health policymaking.

## Conclusion

Our meta-analysis showed that prevalence of HSV2 is significantly higher than that of HSV1. These results provided clear evidences for policy making which is suitable to implement strategies for health promotion. 

## Conflict of interest

All authors declare that there is no conflict of interest.
